# Robusta coffee extracts inhibit quorum sensing activity in *Chromobacterium violaceum* and reduce biofilms against *Bacillus cereus* and *Staphylococcus aureus*

**DOI:** 10.14202/vetworld.2022.2391-2398

**Published:** 2022-10-12

**Authors:** Porwornwisit Tritripmongkol, Suthinee Sangkanu, Ratchadaporn Boripun, Juthatip Jeenkeawpieam, Julalak Chuprom, Veeranoot Nissapatorn, Maria de Lourdes Pereira, Alok K. Paul, Watcharapong Mitsuwan

**Affiliations:** 1 Center of Scientific Equipment for Advanced Research, Thammasat University, Pathumthani 12120, Thailand; 2 School of Allied Health Sciences, Southeast Asia Water Team (SEA Water Team), World Union for Herbal Drug Discovery, and Research Excellence Center for Innovation and Health Products, Walailak University, Nakhon Si Thammarat, Thailand; 3 Akkhraratchakumari Veterinary College, Walailak University, Nakhon Si Thammarat, 80160, Thailand; 4 One Health Research Center, Walailak University, Nakhon Si Thammarat, 80160, Thailand; 5 CICECO-Aveiro Institute of Materials and Department of Medical Sciences, University of Aveiro, Aveiro, 3810-193, Portugal; 6 School of Pharmacy and Pharmacology, University of Tasmania, Hobart, TAS 7001, Australia; 7 Center of Excellence in Innovation of Essential Oil, Walailak University, Nakhon Si Thammarat, 80160, Thailand

**Keywords:** *Bacillus cereus*, biofilms, quorum sensing, Robusta coffee extract, *Staphylococcus aureus*

## Abstract

**Background and Aim:**

*Bacillus cereus* and *Staphylococcus aureus* cause foodborne intoxication in humans and animals. Pathogens can produce biofilms controlled by the quorum sensing system. The study aimed to investigate the antibacterial, antibiofilm, and anti-quorum sensing activities of *Coffea canephora* P. ex Fr. (Robusta coffee) extracts against *B. cereus* and *S. aureus*.

**Materials and Methods:**

Ethanol extracts of fruit peels and seeds of Robusta coffee were tested for antibacterial activity against *B. cereus* and *S. aureus* using a broth microdilution assay. Reduction of the biofilm formation and elimination of the viability of mature biofilm-grown cells of *B. cereus* and *S. aureus* were determined. Inhibition of quorum sensing activity in *Chromobacterium violaceum* by the extracts was investigated using the disk diffusion method and flask incubation assay.

**Results:**

Fresh fruit peel extract showed the strongest antibacterial activity against *B. cereus* and *S. aureus* with minimum inhibitory concentration (MIC) values of 2 and 4 mg/mL, respectively. However, the extracts did not inhibit *Escherichia coli*, avian pathogenic *E. coli*, and *Pseudomonas aeruginosa* at 8 mg/mL. Significant inhibition of biofilm formation at 1/2 × MIC of the fresh peel extract was detected in *B. cereus* (56.37%) and *S. aureus* (39.69 %), respectively. At 8 × MIC of the fresh peel extract, a significant elimination of the mature biofilm viability was detected in *B. cereus* (92.48%) and *S. aureus* (74.49%), respectively. The results showed that fresh and dried peel fruit extracts at 1/2 × MIC significantly reduced violacein production with the highest percentage inhibition ranging from 44.53 to 47.48% at 24 h (p ≤ 0.05).

**Conclusion:**

The results of the present study suggest the potential therapeutic benefits of Robusta coffee extracts in inhibiting the growth, biofilm, and quorum sensing of both *B. cereus* and *S. aureus*. The results put forward an alternative strategy to control the foodborne intoxications caused by both pathogens.

## Introduction

Infectious diseases caused by foodborne and zoonotic pathogens are a major concern worldwide. *Bacillus cereus*, a Gram-positive bacterium, is a well-known causative agent of foodborne intoxication. The toxins produced by *B. cereus* cause food poisoning and results in emetic (vomiting) and diarrheal syndrome [1]. It contaminates rice-based products, fresh vegetables, meat, and dairy products. *Bacillus cereus* also causes mastitis in dairy cows [2] and goats [3]. *Staphylococcus aureus* is a Gram-positive pathogen and a causal agent of several infectious diseases ranging from skin infections to life-threatening. Pathogens are one of the main causes of bovine mastitis in dairy cows and skin infections in animals [4]. *Staphylococcus aureus* and methicillin-resistant *S. aureus* are zoonotic pathogens transmitted from animals to humans [5]. The pathogenesis of *S. aureus* is likely associated with virulence factors, such as structural components, enzymes, toxins, and biofilm [6].

The bacterial biofilm is a community of microorganisms attached and embedded into the extracellular polymeric substance. The biofilm enhances the survival rate of the bacteria in the host by evading the host’s immune system. The biofilms play an important role in the pathogenesis of the infection and are linked to antibiotic resistance [7]. *Bacillus cereus* produces biofilms and secretes various substances such as surfactants, bacteriocins, enzymes, and toxins into the biofilm [8]. A highly resistant and adhesive spore generated by the pathogens further increases the resistance to antimicrobial compounds [8]. *Staphylococcus aureus* infects animals and internalizes bovine mammary epithelial cells to cause mastitis. Biofilm production is an important strategy for *S. aureus* to evade the cow’s immune response [9]. The bacterial biofilm formation is controlled by the quorum sensing system, similar to the production of violacein in *Chromobacterium violaceum* [10]. Quorum sensing is a well-known system of cell-to-cell communication in bacteria and regulates various bacterial virulence factors, including biofilm, swarming, and production of toxins. Quorum sensing enhances bacterial survival, virulence, and resistance to the host immune system and antibiotic resistance [11].

Inhibition of bacterial virulence by inhibiting quorum sensing can be an alternative strategy to overcome infectious diseases. Medicinal plants are mostly used to treat bacterial infections due to their bioactive secondary metabolites. The present study is focused on the antibacterial activities of the extracts of *Coffea canephora* P. ex Fr. (Robusta coffee) belonging to the family Rubiaceae. Robusta coffee is native to Thailand. Coffee is one of the most widely consumed beverages in the world [12]. Several studies have reported the antibacterial activity of extracts from coffee against pathogens [13, 14]. However, studies on the antibacterial activities of Robusta coffee extract against *B. cereus* and *S. aureus* biofilms and biofilm-related quorum sensing are scarce.

This study aimed to investigate the antibacterial and antibiofilm activity of Robusta coffee extracts, including fruit peels and seeds, against *B. cereus* and *S. aureus*. In addition, the anti-quorum sensing activity of the extract against *C. violaceum* was also investigated.

## Materials and Methods

### Ethical approval

The study did not involve any live animals or humans, so ethical approval was not necessary.

### Study period and location

**Figure-1:**
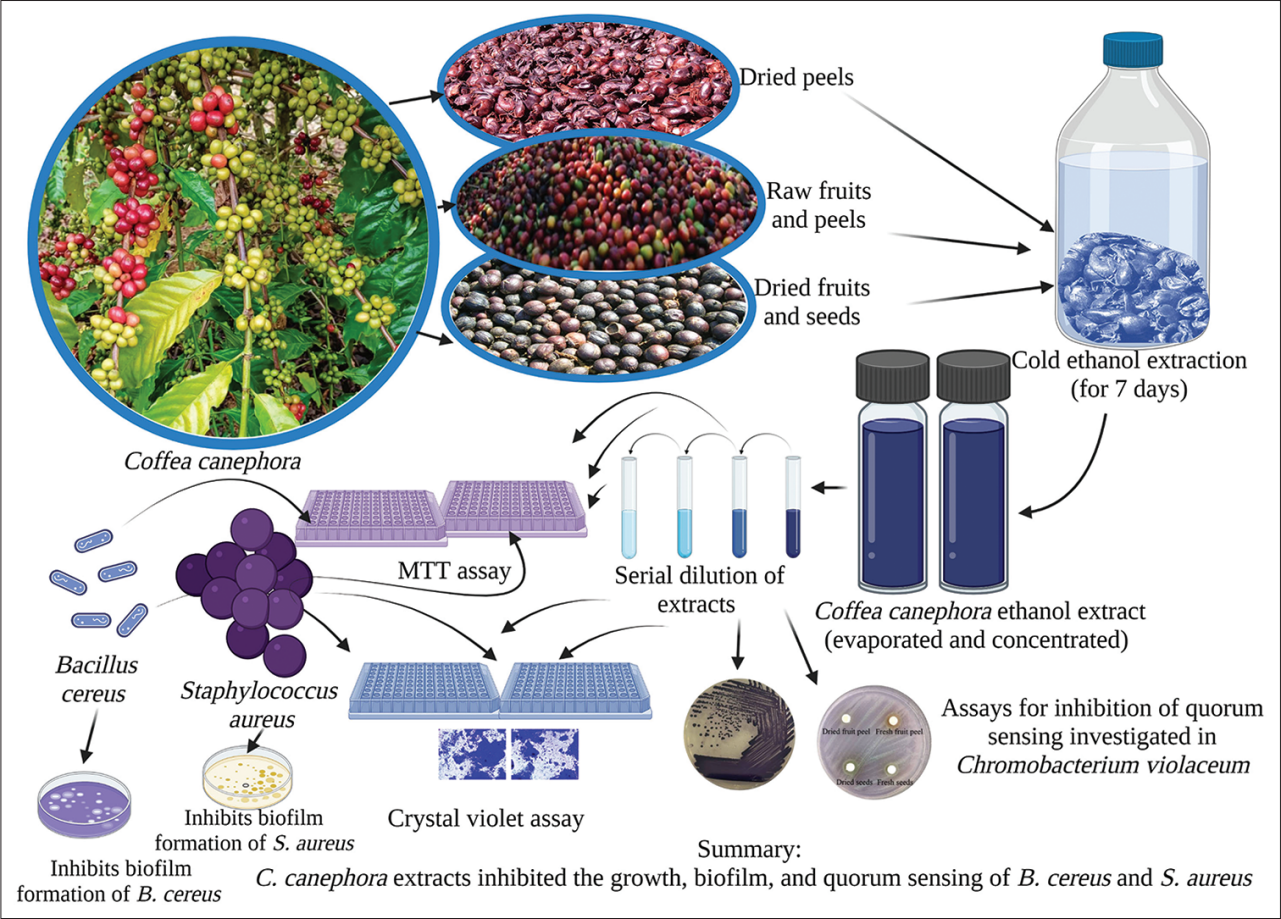
Antibacterial and antibiofilm activities of Robusta coffee extracts, including fruit peels and seeds, against *Bacillus cereus* and *Staphylococcus aureus* are presented. The anti-quorum sensing activity of the extract against *Chromobacterium violaceum*, a biomonitor strain, is elucidated [Source: Biorender.com].

The study was conducted from January 2022 to April 2022. Samples of Robusta coffee were collected from an organic Robusta coffee farm located in Phipun district, Nakhon Si Thammarat Province, South Thailand. Extraction, antibacterial activity tests, antibiofilm activity, and anti-quorum sensing activity of the extracts were investigated at Walailak University, Nakorn Si Thammarat, Thailand. In addition, the overall experiments are described and summarized in Figure-1.

### Bacterial strain and growth condition

*Bacillus cereus* WU22001 was obtained from the Microbiology Laboratory of the Center for Scientific and Technology Equipment, Walailak University, Thailand. Avian pathogenic *Escherichia coli* CH09 (APEC) was kindly obtained from Assistant Professor Dr. Thotsapol Thomrongsuwannakij. *Escherichia col*i ATCC25922, *S. aureu*s ATCC25923, and *P. aeruginos*a ATCC27853 were purchased from the American Type Culture Collection. *Chromobacterium violaceum* DMST21761 was kindly obtained from Assoc. Prof. Dr. Pimonsri Mittraparp-arthorn, Department of Biological Science, Faculty of Science, Prince of Songkla University, Thailand. A single colony of each bacterium from Tryptic Soy Agar (TSA) (Difco, Claix, France) was cultured in Tryptic Soy Broth (TSB) (Difco), incubated at 37°C for 18–24 h, and stored in TSB containing 25% glycerol at −80°C until use.

### Robusta coffee extracts preparation

Fruit peels and fruit seeds of Robusta coffee were used to prepare the crude extracts. Fresh and dried samples of fruit peel and seeds were separately soaked in 500 mL of 95% ethanol for 7 days at 25-30°C. Later, it was filtered and then evaporated under reduced pressure to harvest the crude extracts as described previously [15] with minor modifications. All extracts were dissolved in 100% dimethyl sulfoxide (DMSO) and stored at 4°C for later use.

### Minimum inhibitory concentration (MIC) and minimum bactericidal concentration (MBC) of the extracts against pathogens

The antibacterial activity of the extracts against pathogens, including *B. cereus*, *S. aureus*, *E. coli*, APEC, and *P. aeruginosa* was tested using broth microdilution assay according to the Clinical and Laboratory Standards Institute [16]. Briefly, 3–5 colonies of the bacteria were cultured in Mueller-Hinton broth (MHB) (Difco) and incubated for 3–5 h at 37°C. A volume of 100 μL of the bacterial suspension (1 × 10^6^ colony-forming unit/mL, CFU/mL) was mixed between 80 μL of the medium and 20 μL of serially diluted extracts in a 96-well microtiter plate and incubated at 37°C for 18 h. Vancomycin and ceftriaxone were used as the positive control and 1% DMSO was used as the negative control. The MIC was defined as the lowest concentration of the chemical that inhibited observable growth. The MBC was defined as the lowest concentration of extract required to kill bacteria. All the tests were carried out in triplicate.

### Effects of fruit peel extract on biofilm formation of *B. cereus* and *S. aureus*

The fruit peel extracts were selected to investigate the effect on biofilm production as they showed strong antibacterial activity against both pathogens. The crystal violet assay was used to investigate the effects of extracts on biofilm formation [17] with minor modifications. Briefly, bacteria were cultured in TSB supplemented with 1% glucose, incubated at 37°C for 12–18 h, and diluted to 2 × 10^6^ CFU/mL. The suspensions were added to a 96-well microtiter plate containing 20 mL of sub-inhibitory antibacterial agents and 80 mL of the medium incubated at 37°C for 24 h. The effects of extracts on bacteria growth were evaluated at an optical density (OD) of 600 nm. Then, phosphate-buffered saline (PBS) was used to wash the wells twice. The wells were dried and stained for 30 min with 200 mL of 0.1% crystal violet solution. Then, washed twice with distilled water and air-dried. Biofilms were dissolved in 200 μL of DMSO. To determine the inhibitory action of the extracts, the biofilm formation was measured at an OD of 570 nm by a microtiter plate reader (Thermo-Scientific, Singapore City, Singapore). The relative percentage of biofilm formation was calculated as follows; (mean OD570 of treatment well/mean OD570 of control well) × 100.

Effects of fruit peel extracts of Robusta coffee on the adhesion of the bacteria to glass slides were observed under a light microscope (Nikon, Tokyo, Japan) at 200×. Briefly, the bacteria were cultured in a sterile glass (0.5 cm × 0.5 cm) in the presence of extracts at different concentrations and incubated at 37°C for 24 h. The samples were washed, air-dried, and stained as described above. The morphology of the bacterial adhesion on the glass surface was observed under the light microscope.

### Effects of fruit peel extracts on mature biofilm of *B. cereus* and *S. aureus*

The activity of fresh and dried fruit peel extracts on the mature biofilm of *B. cereus* and *S. aureus* was carried out by 3-(4,5-dimethylthiazol-2-yl)-2,5-diphenyltetrazolium bromide (MTT) assay [17] with minor modifications. Briefly, 200 μL of the bacterial suspension was transferred to a 96-well microtiter plate and cultured at 37°C for 2 days to produce a mature biofilm. The medium was discarded and washed twice with PBS after incubation. Extracts were added to develop biofilms at concentrations of 2–8 × MIC and incubated at 37°C for 24 h. Phosphate-buffered saline supplemented with 10 mL MTT, 5 mg/mL; Sigma-Aldrich, Missouri, USA, was added and the plate was incubated for 2 h at 37°C. 3-(4,5-Dimethylthiazol-2-yl)-2,5-diphenyltetrazolium bromide was cleaved by the dehydrogenase enzyme from live bacterial cells to produce the insoluble purple formazan. Samples were dissolved by 100% DMSO, measured at OD 570 nm. The formulation of viability biofilm percentage was calculated as follows; (mean OD570 of treated well/mean OD570 of control well) × 100.

### Inhibition of quorum sensing activity by the extracts against *C. violaceum*

The inhibition of the quorum sensing activity by Robusta coffee extracts against *C. violaceum*, a biomonitor strain, was carried out by disk diffusion assay and flask incubation assay [18]. Briefly, the overnight culture of the bacterium on TSB was spread on TSA plates. Each extract (10 μl) at a concentration of 100 mg/mL was dropped on a 6 mm paper disk to give a final concentration of 1.0 mg of extract/disk. Then, the disks were placed on plates of bacterial culture and incubated at 37°C for 24 h and the inhibition zone was measured.

The inhibitory activity of the extracts to violacein production was assessed using a flask incubation technique. The bacterium was cultured in Erlenmeyer flasks containing TSB and extracts at different concentrations. The samples were shaken with an agitation speed of 150 rpm at 35°C for 24 h. The bacterial growth in the culture medium was determined by measuring the OD at 600 nm. The bacterial cells were pelleted from the 1 mL culture by centrifugation at 280× *g* for 5 min. The pigment violacein was extracted from the bacteria cells using 100% DMSO and centrifugation at 280× *g* for 5 min. Then, 200 μL of violacein was added to a 96-well microplate and measured OD at 570 nm.

### Effects of the extracts against *S. aureus* staphyloxanthin

The effect of the extracts on *S. aureus* staphyloxanthin was observed qualitatively. Briefly, an overnight culture of *S. aureus* inoculated into TSB was adjusted to McFarland standard number 0.5 in TSB. An aliquot of the bacterial suspension was added to a tube containing the fresh and dried tissue extracts at different concentrations. About 1% DMSO was used as a negative control. The samples were cultured at 37°C for 24 h. Then, the suspension was dropped on TSA and cultured at 37°C for 24 h. The color of the colonies was observed and compared with the control.

### Statistical analysis

All the experiments were performed in triplicate. The results were presented as mean ± standard deviation. All data were recorded and entered using the statistical package software (SPSS Inc., Chicago, IL, USA). The two-tailed unpaired Student’s t-test was used to compare the data between the control and the treatment groups. Differences were considered significant at p ≤ 0.05.

## Results

### Antibacterial activity of Robusta coffee extracts against pathogens

The antibacterial activity of the extracts from different tissues of Robusta coffee was determined and the fresh fruit peel extract demonstrated the strongest antibacterial activity against *B. cereus* and *S. aureus* with MIC values of 2 and 4 mg/mL, respectively (Table-1). The dried fruit peel extracts and dried seed extract showed antibacterial activity against both pathogens at 4–8 mg/mL. However, the extracts did not show antibacterial activity against the tested Gram-negative bacteria, including *E. coli*, APEC, and *P. aeruginosa* at 8 mg/mL.

**Table-1:** Minimum inhibitory concentration (MIC) and minimum bactericidal concentration (MBC) of the extracts from different parts of Robusta coffee against the pathogens.

Pathogens	MIC/MBC (mg/mL)				Antibiotics

	Fresh fruit peel	Dried fruit peel	Fresh seeds	Dried seeds	
*B. cereus*	2/4	8/>8	>8/>8	8/>8	0.001/0.002^a^
*S. aureus*	4/>8	4/>8	>8/>8	8/>8	0.001/0.002^a^
APEC CH09	>8/>8	>8/>8	>8/>8	>8/>8	0.00025/0.00025^b^
*E. coli*	>8/>8	>8/>8	>8/>8	>8/>8	0.00025/0.00025^b^
*P. aeruginosa*	>8/>8	>8/>8	>8/>8	>8/>8	0.001/0.002^b^

a Vancomycin,

b Ceftriaxone

### Antibiofilm formation activity of the extracts against *B. cereus* and *S. aureus*

**Figure-2:**
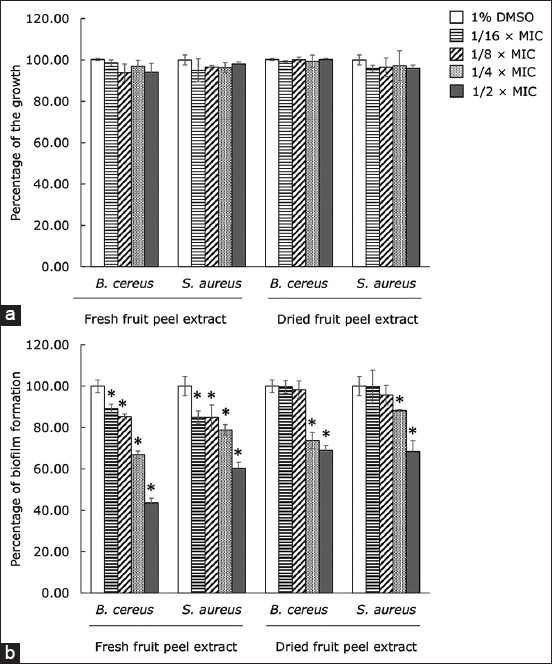
(a) Effects of extracts from different parts of Robusta coffee on the growth and (b) the biofilm formation of *Bacillus cereus* and *Staphylococcus aureus*. The pathogens were grown in Tryptic Soy Broth plus 1% glucose and the extracts at sub-minimum inhibitory concentrations. About 1% dimethyl sulfoxide was used as a negative control. The relative percentage of the biofilm formation was defined as: (mean OD570 of treated well/mean OD570 of control well) × 100. The result was presented as mean ± standard deviation (*significant difference; p ≤ 0.05).

The fruit peel extract of Robusta coffee was chosen to investigate the antibiofilm activity against *B. cereus* and *S. aureus* because it performed best among other tested extracts against the pathogens. It is important to emphasize that the tested concentration of extracts did not inhibit the growth of both pathogens (Figure-2a). As shown in Figure-2b, both fresh fruit peel extract and dried fruit peel extract exhibited significant antibiofilm activity with a dependent concentration against both pathogens (p ≤ 0.05). Using 1/2 × MIC of the fresh peel extract, 56.37 and 39.69% inhibition of the biofilm formation were observed in *B. cereus* and *S. aureus*, respectively. In addition, 31% inhibition of the biofilm was found in both pathogens when the bacterial cells were treated with dry peel extract at 1/2 × MIC, compared to the control.

**Figure-3:**
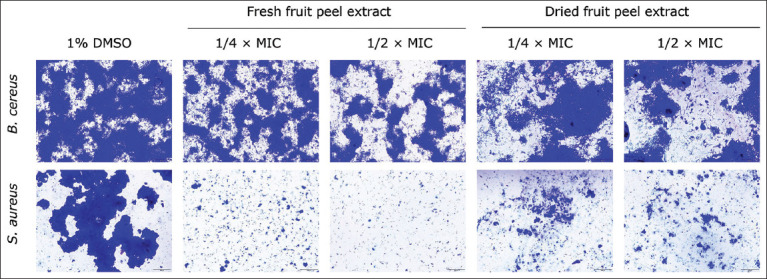
Adhesion of *Bacillus cereus* and *Staphylococcus aureus* cells in the biofilms in the presence of sub-minimum inhibitory concentrations (MICs) of Robusta coffee extracts observed by light microscope. The bacteria were cultured in Tryptic Soy Broth plus 1% glucose and the extracts at 1/4 × MIC and 1/2 × MIC. One percent dimethyl sulfoxide was used as a negative control. Magnifications were observed at 200X.

Adhesion is the first step in biofilm formation [15]. It was found that both *B. cereus* and *S. aureus* treated with fresh and dried peel extracts showed a reduction in the adhesion on the glass slide, as observed by a light microscopy (Nikon, Tokyo, Japan) (Figure-3).

### Elimination of mature biofilm of *B. cereus* and *S. aureus* by the extracts

**Figure-4:**
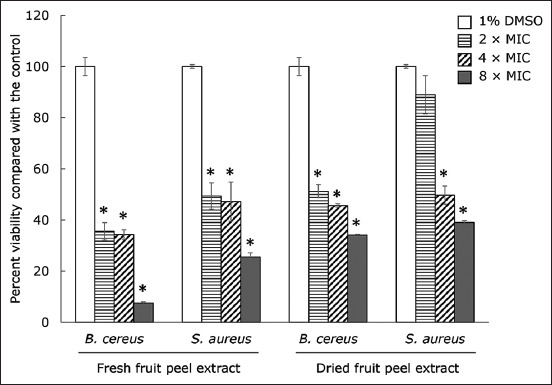
Activity of Robusta coffee extracts on established biofilm of *Bacillus cereus* and *Staphylococcus aureus*. The bacteria were grown in the medium to produce mature biofilms on 2^nd^ day. The biofilm was treated with extracts at different concentrations. About 1% dimethyl sulfoxide was used as a negative control. The relative percentage of biofilm viability was defined as: (mean OD570 of treated well/mean OD570 of control well) × 100. The data were presented as mean ± standard deviation (*significant difference; p ≤ 0.05).

A significant elimination in the viability of both mature biofilm-grown cells of *B. cereus* and *S. aureus* was observed on treatment with the fresh and dried fruit peel extract at 2 × MIC, compared to the control (p ≤ 0.05) (Figure-4). At 8 × MIC of the fresh peel extract, 92.48 and 74.49% of the elimination of the mature biofilm viability were detected in *B. cereus* and *S. aureus*, respectively. In addition, at 8 × MIC, the dried fruit peel extract showed 65.93 and 61.01% elimination in viability against the mature biofilm of *B. cereus* and *S. aureus*, respectively.

### Anti-quorum sensing activity of Robusta coffee extracts against *C. violaceum*

**Figure-5:**
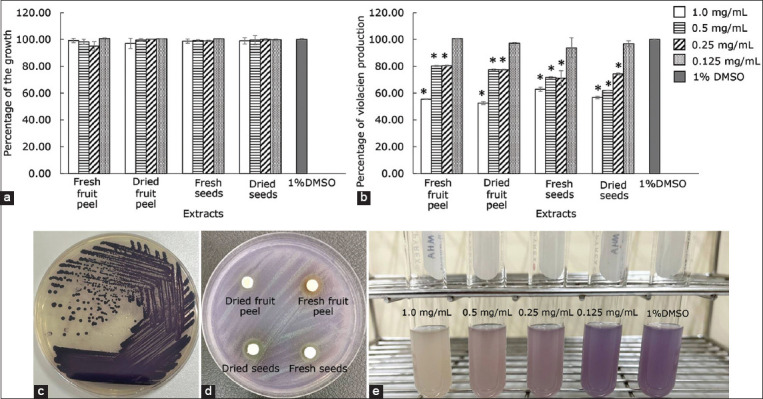
(a) Effects of Robusta coffee extracts on the growth and (b, d, and e) quorum sensing activity in *Chromobacterium violaceum*, a biomonitor strain. (c) The bacterium produces a violacein pigment that is controlled by the quorum sensing system, resulting in a violet colony. About 1% dimethyl sulfoxide was used as a negative control. Inhibition of the quorum sensing system by the extracts showed a decrease in pigment production without growth.

Inhibition of the violacein pigment by the extracts without the growth of the bacteria was considered the anti-quorum sensing activity (Figure-5c). The result demonstrated that all extracts, including fresh fruit peel extract, dried fruit peel extract, fresh seed extract, and dried seed extract, showed anti-quorum sensing ability at the concentration of 1.0 mg/disk (Figure-5d). The quorum sensing inhibition zones of the extracts ranged from 8.33 ± 0.58 to 10.00 ± 1.73 mm (Table-2). The fresh fruit peel extract showed the largest zone with 10.00 ± 1.73 mm.

**Table-2:** Anti-quorum sensing activity of the extracts from different parts of Robusta coffee against violacein production produced by *C. violaceum.*

Bacterium	Anti-quorum sensing activity (mm)			

	Fresh fruit peel	Dried fruit peel	Fresh seeds	Dried seeds
*C. violaceum*	10.00±1.73	8.33±0.58	9.33±1.53	9.33±0.58

The inhibitory activity of the extracts was determined by quantifying violacein production at OD 570 nm. The reduction in violacein production by the extracts was concentration-dependent (Figure-5b). The results showed that the fresh and dried fruit peel extracts at 1/2 × MIC significantly reduced violacein production with the highest percentage of inhibition ranging from 44.53 to 47.48% at 24 h (p ≤ 0.05). The inhibition of violacein production in *C. violaceum* by the fresh fruit peel extract was also concentration-dependent (Figure-5e). No significant inhibition of bacterial growth in comparison to control was observed (Figure-5a).

### Effects of Robusta coffee extracts on staphyloxanthin in *S. aureus*

**Figure-6:**
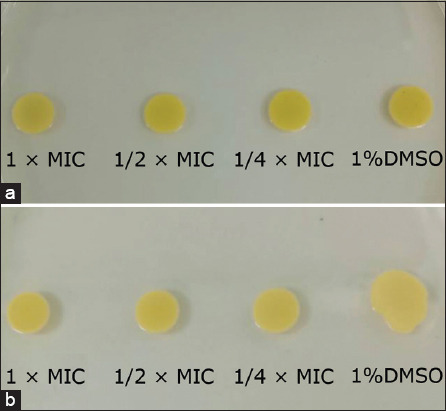
Effects of Robusta coffee extracts on staphyloxanthin production in *S. aureus*. (a) The bacterium was treated with fresh and (b) dried extracts at different concentrations for 24 h. About 1% dimethyl sulfoxide was used as a negative control. Then, the suspension of the bacterium was dropped on Tryptic Soy Agar. The color of the colonies was observed and compared with the control.

Staphyloxanthin is a yellow pigment and a virulence factor produced by *S. aureus*. The activity of Robusta coffee extracts on staphyloxanthin in *S. aureus* was investigated by incubating the bacterium in the medium supplemented with the extract, followed by culture on the agar plate. The result demonstrated that the fresh fruit peel extract and the dried fruit peel extract did not inhibit staphyloxanthin production in *S. aureus*, compared to the control (Figure-6a and b).

## Discussion

Coffee is one of the most widely consumed beverages in the world. The use of natural products as part of a daily diet may be a more effective and safer alternative for disease prevention [19]. We investigated the antibacterial and antibiofilm activities of Robusta coffee extracts against *B. cereus* and *S. aureus*, causative agents of foodborne bacterial intoxication.

*Bacillus cereus* and *S. aureus* are zoonotic pathogens [5]. The bacterial transmission from close contact between household pets and people has been reported [20]. Furthermore, livestock-associated MRSA responsible for human colonization and infection has also been described [21]. The peel extracts of Robusta coffee exhibited a bacteriostatic effect against *B. cereus* and *S. aureus* due to the MBC/MIC ratio of the extracts against pathogens [15]. Similarly, the aqueous extracts from the pulp of Arabica coffee (*Coffea arabica* L.) have also shown bacteriostatic effects against *S. aureus*, *S. epidermidis*, *P. aeruginosa*, and *E. coli* [22]. It has been reported that phenolic and flavonoid compounds were detected in the fresh peel of Robusta coffee which could be responsible for the bacteriostatic effect [23]. Inhibition of the cell membrane function and energy metabolism was considered as possible mechanisms of action of the flavonoid against the bacteria [24]. In addition, phenolic compounds denature bacterial cell proteins and inhibit cell multiplication [25]. The extract of the coffee peels demonstrated antioxidant activity due to the presence of phenolic contents in the extract [22]. The Gram-positive bacteria showed higher susceptibility to extracts than Gram-negative bacteria. The reason may be due to the different composition of the bacterial cell envelope in the Gram-negative and positive bacteria, the latter lacking a barrier of low permeability like the outer membrane [13]. In addition, some hydrophobic compounds such as phenols and tannins are difficult to absorb into the outer membrane (composed of phospholipids) of the Gram-negative pathogens [22].

Biofilms in *B. cereus* and *S. aureus* play a crucial role in the pathogenicity and evading the host immune system. The bacterial biofilm production is controlled by the quorum sensing system [10]. Quorum sensing is a bacterial cell-to-cell communication process and regulates bacterial virulence. In *B. cereus*, quorum sensing is used to establish infections by producing an arsenal of virulence factors, including enterotoxins, pore-forming hemolysins, cytotoxins, and various degradative enzymes. The quorum sensing system in *B. cereus* utilizes an autoinducing PapR peptide signal that mediates the activation of the pleiotropic virulence regulator PlcR, a 34 kDa protein that acts as a bacterial virulence transcription factor [26]. In addition, the regulatory network that controls the biofilm formation in *B. cereus* is by the induction of a SinR regulator that binds to SinI [8]. Consequently, the complex molecule of the SinI/SinR pair acts as a switch between biofilm formation and swimming motility in the pathogen [8]. An accessory gene regulator (*agr*) of the quorum sensing system plays a crucial role in regulating *S. aureus* biofilm formation [27].

The present study demonstrated that extracts of Robusta coffee act as the inhibitor of the biofilm formation by *B. cereus* and *S. aureus* and quorum sensing activity in *C. violaceum*. Our result showed that the anti-quorum sensing activity of the coffee extracts in *C. violaceum* resulted in the reduction of violacein pigment. The downregulation of *las I* and *las R* virulence-associated genes in *P. aeruginosa* was detected after treatment with coffee extract [28]. Inhibition of *las I* and *las R* in *P. aeruginosa* resulted in inhibition of biofilm and elastase production [29]. Similarly, the quorum sensing inhibitor RNAIII-inhibiting peptide (RIP) inhibited *S. aureus* TRAP/*agr* systems and the biofilm produced by *S. aureus* [30]. However, inhibition of staphyloxanthin was not observed after treatment of bacterial cells with Robusta coffee extract.

The isolation of pure compounds from Robusta coffee extract should be determined in future studies. Subsequently, antibacterial modes of action of the extract and its pure compounds against the pathogens need to be investigated. Further, the *in vivo*/*ex vivo* studies using the pure compounds are required to provide insight into the antimicrobial action of Robusta coffee extracts against both *B. cereus* and *S. aureus*.

## Conclusion

The present study revealed the bacteriostatic effect of Robusta coffee extracts against *B. cereus* and *S. aureus*. Fresh fruit peel extract demonstrated the strongest antibacterial activity against *B. cereus* and *S. aureus* with MIC values of 2 and 4 mg/mL, respectively. Fresh and dry peel extracts significantly reduced the biofilm formation in both *B. cereus* and *S. aureus*, compared to the control. At 8 × MIC of the fresh peel extract, 92.48 and 74.49% of the elimination of the mature biofilm viability were detected in *B. cereus* and *S. aureus*, respectively. The results showed that the fresh and dry extracts of the fruit peels significantly reduced the violacein production in *C. violaceum* within 24 h. The results suggested the potential medicinal benefits of Robusta coffee extracts in inhibiting growth, biofilm production, and quorum sensing in both pathogens (*B. cereus* and *S. aureus*).

## Authors’ Contributions

WM, PT, SS, JJ, and VN: Conceived and designed the experiments. WM, SS, JC, JJ, and RB: Performed the experiments. PT, SS, RB, AKP, JJ, and WM: Analyzed and interpreted the data. WM and JJ: Statistical analysis. PT, VN, AP, and MLP: Contributed reagents, materials, analysis tools, or data. WM, RB, JJ, and VN: Wrote the paper. All authors have read and approved the final manuscript.
